# Enhancing Rehabilitation and Healthcare Training Using Augmented Reality: Lessons From the AREduX Prototype

**DOI:** 10.7759/cureus.114022

**Published:** 2026-08-05

**Authors:** Eva Peisachovich, Bill Kapralos, Celina Da Silva, Shai Rahmanov, Kayla Jachecki

**Affiliations:** 1 Medical Education and Simulation, York University, Toronto, CAN; 2 Faculty of Information Technology/Health Education Technology Research Unit, Ontario Institute of Technology, Oshawa, CAN; 3 Health Research, York University, Toronto, CAN

**Keywords:** augmented reality (ar), education, healthcare, rehabilitation, simulation

## Abstract

Augmented reality (AR) is increasingly recognized as a valuable tool in healthcare education and rehabilitation. The Augmented Reality Education Experience (AREduX) is a prototype designed to simulate the physical and cognitive symptoms of dementia, enhancing empathy and understanding among healthcare professionals and caregivers of people living with dementia (PLWD).

Extrapolating from what we learned from focus-group interactions with study participants, AREduX usability testing, NASA Task Scale, and prototype implementation into an educational context, this paper presents possible applications of AREduX beyond the PLWD population. It describes how AREduX can be used beyond dementia care for broader applications in rehabilitation, competency-based education, and experiential training for physical and rehabilitation scenarios.

## Editorial

Introduction

This perspective paper is about how the application of Augmented Reality Education Experience (AREduX) can be utilized beyond dementia care. Technological advancements in simulation-based training have significantly transformed healthcare education and rehabilitation. Among these advancements, augmented reality (AR) stands out for its ability to create immersive, experiential learning environments by overlaying digital elements onto the real world. AREduX is a proof-of-concept prototype designed to support the development of empathy among healthcare providers and caregivers of persons living with dementia (PLWD). AREduX simulates the physical, sensory, and cognitive challenges faced by PLWD to foster empathetic caregiving interactions and enhance caregiving competencies.

This editorial illustrates the findings supported by AREduX testing and presents future applications from what we learned as potential future directions. The development and evaluation of AREduX followed a structured, multi-phased process to ensure its effectiveness and usability.

Development and Design

The AR experience was developed based on insights from focus groups and expert consultations, ensuring that it effectively addresses the real-world challenges of caregiving for PLWD [1]. Usability testing and prototype iterations: The initial prototype was tested to evaluate its usability and effectiveness, and iterative refinements were implemented to enhance functionality and user experience [2-7].

Refined Prototype Testing in an Educational Context and Evaluation

AREduX's impact in an educational setting was assessed by measuring changes in participants' empathy levels before and after engaging with the simulation.

This structured approach was intended to provide caregivers with a deeper understanding of the experiences of PLWD, support the development of empathy, and ultimately improve care and patient satisfaction.

Simulation scenario and design

AREduX currently focuses on a single simulation scenario set in a typical home environment, where users interact with everyday objects. The scenario involves setting a table for dinner (e.g., placing a plate, knife, and fork), making a sandwich, and eating it. This scenario was chosen based on feedback from a focus group of stakeholders, including experts in cognitive health and aging and professionals with expertise in caring for PLWD and their caregivers. The tasks were chosen to represent the common daily activities that may become challenging for PLWD.

Although the initial focus is on this one scenario, the AREduX platform was designed with sufficient flexibility to support other scenarios and modifications to existing experiences. The user interface is designed to be simple and graphically based, allowing healthcare educators with limited programming knowledge to create and modify scenarios with minimal technical expertise.

**Figure 1:**
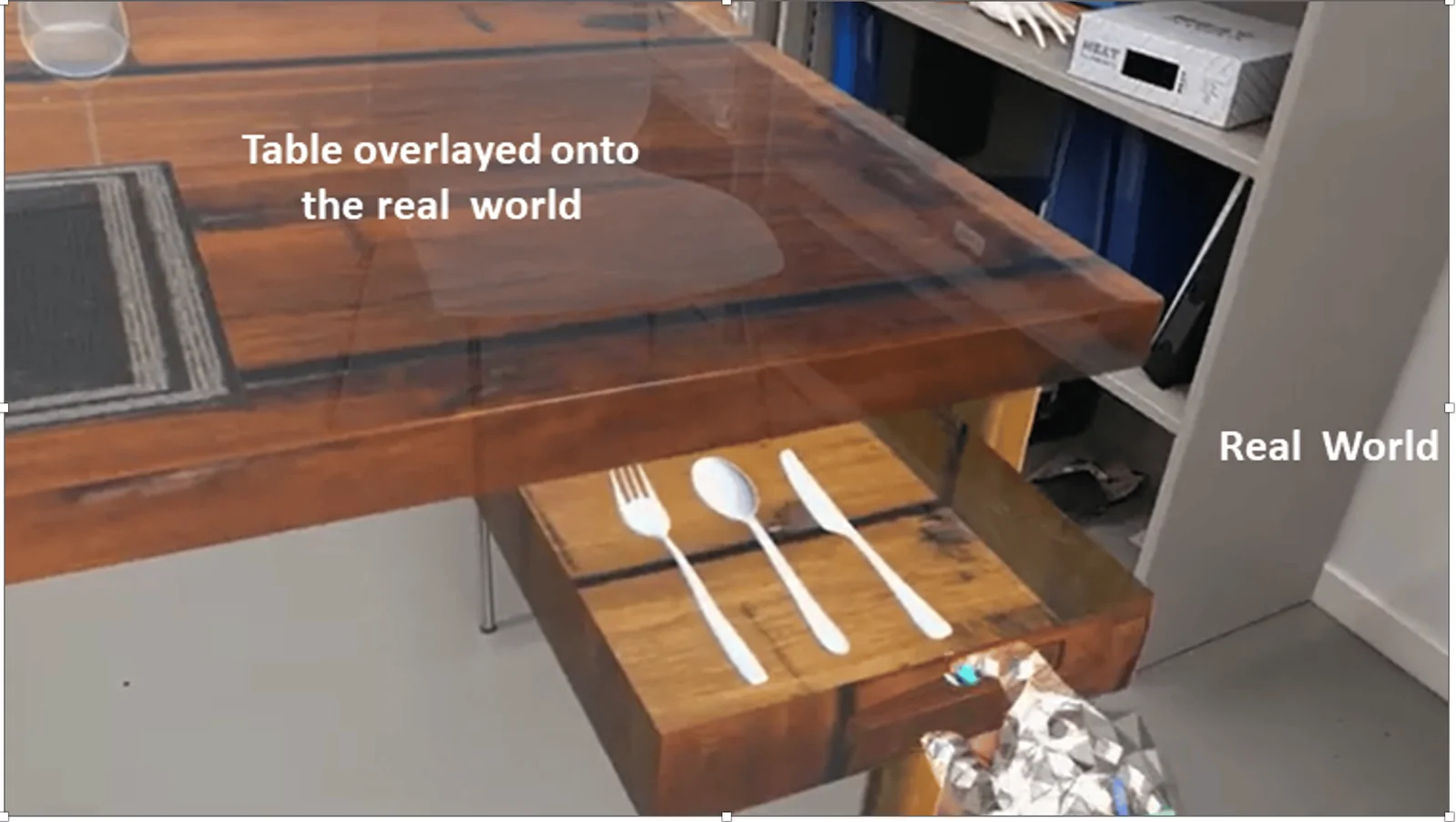
Screenshot of the scenario during a test session where the table is overlaid onto the real world

A sample screenshot of the simulation (Figure 1) illustrates the computer-generated table overlaid onto the real world (in this case, a university laboratory). The user, wearing the Microsoft AR HoloLens 2 mixed-reality headset (Microsoft Corporation, Redmond, WA, US), interacts with the virtual environment by opening a drawer and removing the cutlery, thereby supporting immersion in the simulated tasks.

The AREduX framework offers a unique opportunity to examine healthcare training and rehabilitation through an interactive, skill-based perspective that emphasizes movement, coordination, and learning. The tasks embedded in AREduX - grasping, placing, and cutting - require accurate hand-eye coordination, controlled use of force, and spatial awareness. These actions allow researchers and educators to observe how users plan, execute, and adapt their movements during task performance, providing valuable insight into motor-skill development and functional engagement.

By simulating object interaction in a controlled AR environment, learners can receive immediate visual and task-based feedback on their interactions and performance, which are key factors in motor learning and functional rehabilitation [8,9]. From a physical theory perspective, repeated engagement with these tasks may reinforce motor patterns through practice and repetition. For example, grasping objects of varying sizes and weights provides opportunities to adjust force output and joint coordination, directly aligning with rehabilitation goals for fine and gross motor-skill recovery [10]. The simulations also enable the study of cognitive-motor integration, as users must plan, execute, and adapt movements in response to dynamic virtual stimuli reflecting challenges encountered by individuals recovering from stroke, living with Parkinson's disease, or experiencing other conditions associated with motor impairments [11].

By combining high usability and immersive user experience with these physically grounded task designs, AREduX creates an environment that not only supports experiential learning for healthcare professionals but may also provide a foundation for future therapeutic and rehabilitation-oriented applications. The platform's modularity allows for task customization, enabling the exploration of additional physical parameters such as grip stability, motion smoothness, or bilateral coordination, which could be measured and analyzed through future system enhancements.

This integration of physical theory into virtual simulations can support competency-based education, evidence-informed rehabilitation practices, and facilitate the transfer of learned skills to real-world clinical and caregiving contexts.

Ultimately, AREduX bridges theoretical principles of physics and motor control with applied healthcare training, demonstrating how immersive simulation can be leveraged to improve both practitioner competence and patient outcomes across diverse clinical populations.

Task performance in physical therapy

**Figure 2:**
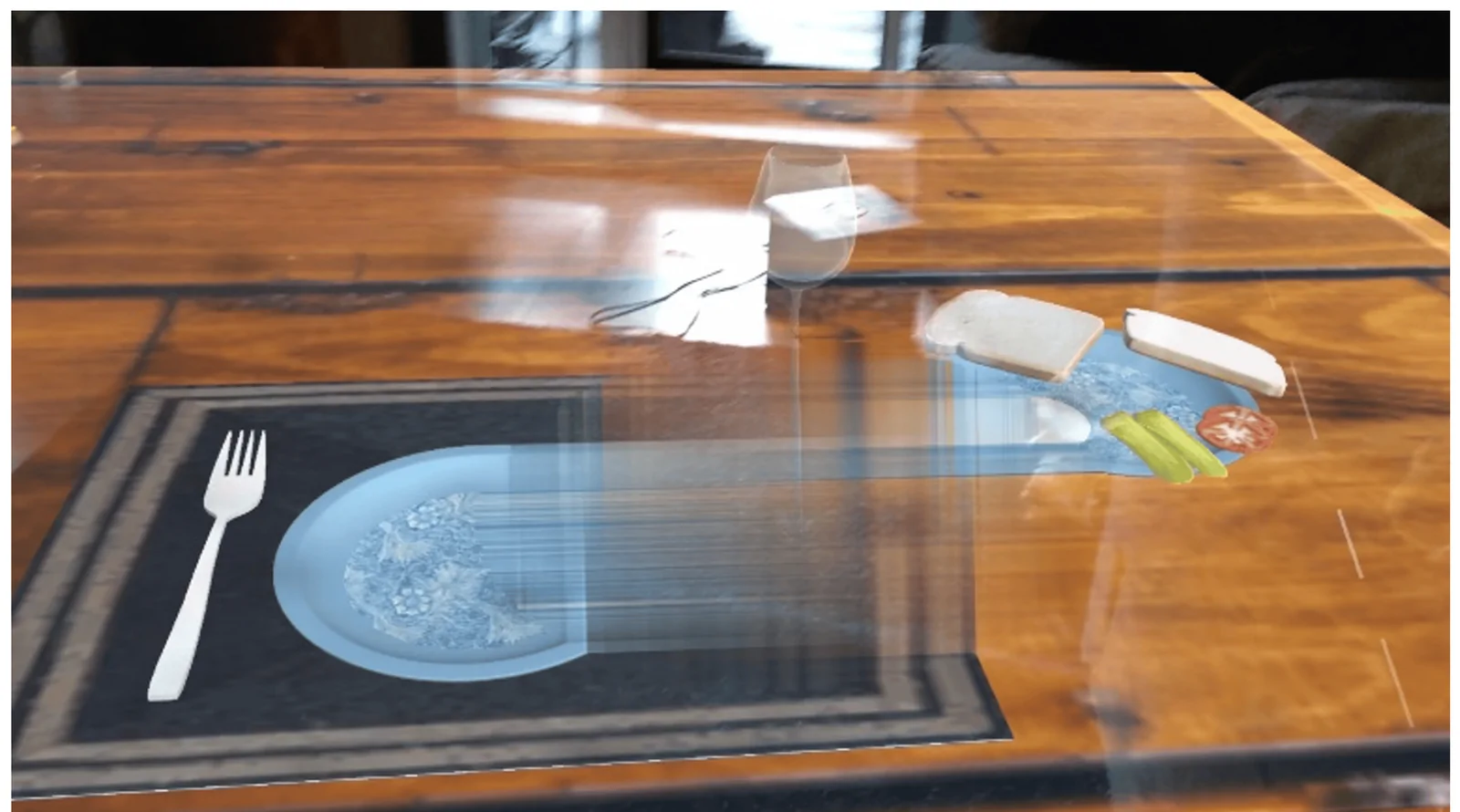
Scenario illustrating a blurred area that is aligned with the user’s eye gaze, simulating macular degeneration

Dementia affects sensory, cognitive, and motor function, creating challenges for PLWD in performing everyday activities [12]. AREduX simulates aspects of these challenges through audiovisual distortions and motor dysfunctions, giving healthcare professionals and physical therapists an insight into or a realistic understanding of the obstacles PLWD face. Visual distortions, such as blurred visuals, reduced contrast, and spatial disorientation, mirror common visual deficits documented in persons living with dementia [13] (Figure 2), while auditory challenges, including sound distortion and background noise, replicate difficulties in communication and environmental awareness [14]. Motor impairments, including delayed or uncoordinated movements, simulate tremors, stiffness, or muscle weakness that are characteristic features of dementia-related motor dysfunction [15], making tasks such as grasping, placing, and cutting more difficult.

In physical therapy, AREduX may provide a platform to examine how these sensory and motor impairments affect task performance and functional movement. By simulating real-life challenges, therapists can design targeted interventions to improve fine and gross motor skills, coordination, and adaptive strategies [10]. The immersive experience could allow practitioners to practice assessing functional limitations, developing patient-specific therapy plans, and exploring approaches to support rehabilitation outcomes. Ultimately, AREduX could bridge experiential learning with physical therapy practice and could support evidence-informed interventions and enhance clinician preparedness [8].

Supporting competency-based education and problem-solving for healthcare professionals

AREduX could contribute to competency-based education for healthcare professionals, as it supports the development of practical, task-based skills that are critical in physical therapy and rehabilitation [16]. The platform creates interactive simulations that could be used to represent real-world clinical scenarios, presenting dynamic, evolving problems that healthcare providers must address during task performance. For example, by simulating impairments in motor coordination, grip strength, and movement timing, the platform allows healthcare professionals and therapists to observe how sensory and motor deficits impact daily activities. Tasks such as grasping, placing, and cutting objects provide opportunities to practice assessing functional limitations, designing adaptive interventions, and reinforcing motor-learning principles [9]. By placing users in simulated patient-centered scenarios, AREduX encourages healthcare professionals to make critical decisions and adjust care strategies as patient conditions change. This hands-on, immersive approach may support clinicians in translating theoretical knowledge into actionable strategies and practicing thinking under pressure, as they would in a real clinical setting, enhancing both patient care and rehabilitation outcomes [8].

Moreover, by simulating evolving patient states, AREduX can help healthcare providers build adaptability, collaboration skills, and key competencies in healthcare [16]. For example, they could interact with simulated healthcare stakeholders and AI-driven virtual characters, reinforcing effective teamwork and communication in fast-paced, decision-driven contexts [17]. Notably, immediate feedback is a core feature of the system, allowing users to evaluate the outcomes of their decisions and refine their strategies for improved patient care [9]. Through repeated simulations, healthcare professionals may receive opportunities to strengthen their ability to assess and manage complex cases, adapt to unexpected complications, and improve their clinical decision-making under uncertainty [8].

Furthermore, the modular design of AREduX enables the customization of tasks to address a wide range of clinical contexts beyond dementia, including post-stroke recovery, geriatric care, pediatric therapy, and neurodevelopmental or motor impairments. By integrating realistic, scenario-based simulations into competency-based education, AREduX can support cognitive and technical skills while strengthening clinicians' confidence and preparedness to provide patient-centered, effective care across diverse populations [16].

Expansion of AREduX for other populations

While AREduX was initially developed with dementia care in mind, its modular and adaptive framework could support significant potential across a wide range of healthcare populations. The platform can be modified to simulate diverse physical, cognitive, or sensory impairments, enabling healthcare professionals to better understand the unique challenges faced by different patient groups. For example, tasks can be adapted to target fine-motor coordination, bilateral hand use, or cognitive load in individuals recovering from stroke, supporting post-stroke rehabilitation and functional recovery [11]. Moreover, simulations can be tailored for geriatric populations to address age-related motor or sensory decline [18], or for children with developmental disabilities to support motor-skill acquisition and cognitive engagement in pediatric therapy.

Beyond physical and cognitive rehabilitation, AREduX could be extended to mental health scenarios or neurodevelopmental conditions [19], offering healthcare professionals experiential learning opportunities to develop empathy, communication, and problem-solving skills with these populations. This adaptability could allow AREduX to be a versatile, scalable tool for competency-based education, rehabilitation planning, and interprofessional training, with applications that could be capable of improving healthcare outcomes across multiple clinical contexts [16].

The platform's modular design also enables task customization to address specific cognitive, sensory, or motor challenges across different patient populations, broadening its potential applications in healthcare education and training.

The adaptability of AREduX makes it a valuable tool for exploring task performance under various conditions. By modifying tasks to reflect the needs of different patient populations, such as incorporating knife-and-fork tasks to assess fine-motor coordination or introducing more complex cognitive challenges, AREduX can support a broader range of healthcare training needs. This flexibility allows the platform to address specific cognitive or physical impairments across multiple disciplines, further enhancing its potential to train and inform healthcare professionals and caregivers about the diverse needs of their patients.

By immersing healthcare providers and caregivers in the simulated experiences of PLWD, AREduX is intended to foster a deeper understanding of the emotional, sensory, and physical difficulties these individuals face. This experiential-learning approach supports the development of empathy, compassion, and practical insight, enabling caregivers and healthcare providers to develop more effective, patient-centered care strategies [1,2]. Experiencing cognitive and sensory impairments firsthand allows users to gain crucial perspectives on the challenges of daily living, which can directly inform communication approaches and intervention planning. As a result, this immersive simulation may contribute to improved caregiving interactions and intervention planning through improved caregiver-patient interactions and tailored therapeutic interventions.

Further, AREduX represents a promising platform for enhancing healthcare professional may provide opportunities for healthcare providers and caregivers to understand the specific obstacles encountered by PLWD and adapt their rehabilitation approaches accordingly [12,13,15]. Through its adaptable, modular design, AREduX can be expanded to simulate a wide range of impairments across various healthcare contexts, such as post-stroke rehabilitation, pediatric therapy, and geriatric care [11,18]. This flexibility allows AREduX to support competency-based education and future rehabilitation-oriented applications, supporting skill development, empathy building, and rehabilitation outcomes.

Feedback from healthcare providers indicates that the functional clarity of the AR experience, including its intuitive overlay, provides a solid foundation for real-world applications in clinical training. However, suggestions for improvements in navigation and interface responsiveness highlight opportunities for optimization. Despite this, there is strong consensus among the participants that AREduX could be integrated into healthcare curricula to support caregiver preparedness and expand its impact beyond dementia care. Ultimately, AREduX stands as a scalable, immersive platform with the potential to address knowledge gaps in healthcare training, particularly in the rehabilitation and care of diverse patient populations.

AREduX in rehabilitation: enhancing motor skill development

AREduX not only serves as an educational tool but also demonstrates potential for future rehabilitation-oriented applications.

VR, AR, and XR simulations are increasingly being used in rehabilitation settings to support individuals with cognitive and physical impairments [20]. The interaction mechanics embedded within AREduX may provide a foundation for exploring how immersive simulations can be adapted to support motor and cognitive training. By simulating real-world challenges, patients can practice tasks in a controlled, supportive environment aligning with principles commonly employed in rehabilitation programs [9].

AREduX also contains several interaction elements that align with objectives commonly found in occupational therapy and motor rehabilitation. The platform's core tasks - grasping, placing, and cutting - are closely aligned with key objectives in occupational therapy and motor rehabilitation, focusing on fine motor coordination, hand-eye coordination, and grip strength. These functional movements are essential for daily activities and are commonly targeted in rehabilitation programs across diverse populations.

The platform therefore provides opportunities to explore how such interactions might be incorporated into future rehabilitation-focused applications.

These features may be relevant to post-stroke rehabilitation, where restoring upper-limb dexterity and grip control is a primary goal [21,22]. Future adaptations of AREduX could incorporate rehabilitation-specific task designs and performance metrics to support targeted intervention development. Similarly, in geriatric care, where age-related motor decline can severely impact daily independence [18], AREduX may provide a platform for developing engaging task-based activities aimed at supporting motor function and functional participation.

Adaptability and future potential

AREduX's adaptability is a key strength in extending its application to a range of rehabilitation contexts. For instance, by incorporating specific tasks, such as knife-and-fork coordination exercises, the platform could be adapted to support the assessment and training of fine motor control, neuroplasticity-driven recovery, and cognitive load, particularly for post-stroke therapy [22]. By simulating sensory overload experiences, AREduX may have potential applications in educating and training caregivers to develop responsive intervention strategies for individuals with sensory processing disorders, autism spectrum disorder, or other neurodevelopmental conditions [19].

These applications build upon the platform's existing modular architecture and task-based interaction design.

Beyond these current capabilities, future iterations may incorporate artificial intelligence (AI)-driven adaptation mechanisms, enabling task parameters and levels of support to be adjusted automatically based on user performance; AREduX could therefore evolve to provide adaptive feedback and personalized task progression, allowing rehabilitation tasks to adapt based on a patient's progress and therapeutic needs [23]. This has the capability to support other personalized rehabilitation experiences for individuals with physical and cognitive impairments.

Real-world applications and research validation

Research supports the efficacy of simulation-based training in improving clinical decision-making, critical thinking, and adaptability [8]. Studies have illustrated that healthcare providers who are trained through simulations demonstrate superior performance in actual clinical situations, particularly in high-pressure environments such as emergency medicine and surgery [8,9]. For example, nurses who undergo simulation training demonstrate improved decision-making abilities in real-world clinical contexts compared to those who receive traditional education [24].

With its ability to support experiential learning and its potential for future rehabilitation-oriented applications, AREduX stands as a versatile, scalable platform that may contribute to the development of skills relevant to healthcare practice.

Through dynamic, immersive learning experiences, AREduX has the potential to support healthcare professionals in understanding the challenges faced by diverse patient populations and in developing strategies for patient-centered care. Future adaptations of AREduX may further extend its application to rehabilitation, geriatrics, pediatric care, and other healthcare contexts. 

While AREduX illustrates considerable promise, several practical barriers may limit its broader implementation in healthcare education and rehabilitation settings. The cost of AR hardware, such as the Microsoft HoloLens 2 headset, represents a significant financial barrier for many institutions, particularly those in under-resourced or community-based settings.

Technical expertise required for scenario development and system maintenance may restrict accessibility, as healthcare educators with limited programming knowledge may face challenges adapting the platform despite its graphically based interface. Moreover, the current evidence base for AREduX remains preliminary, grounded in small-scale usability testing and focus group data; larger, controlled studies are needed before its educational and rehabilitation outcomes can be generalized across diverse clinical populations.

Lastly, user familiarity with AR technology varies, and there may be learners or clinicians who may require dedicated orientation and training before engaging meaningfully with the simulation, making the implementation a burden. Addressing these barriers through institutional investment, interprofessional technical support, and iterative user-centered design will be imperative to realizing AREduX's scope as a scalable tool in healthcare training and rehabilitation.

Future recommendations

The findings from usability testing, focus group interviews, and NASA Task Load Index assessments [25] of the prototype provide several design and educational recommendations for the continued development and application of AREduX.

First, participants consistently emphasized the importance of interface simplicity. Based on these findings, future iterations of AREduX should maintain a clutter-free interface that provides intuitive access to tools, learning resources, and simulation modules. Participants reported that limiting visual and interactive elements to those directly relevant to the task improved focus, reduced distractions, and supported task completion.

Second, usability testing and NASA-TLX results highlighted the importance of balancing cognitive and physical demands during task performance. Participants reported higher engagement when tasks were appropriately challenging but not overwhelming. Consequently, future versions of AREduX should incorporate mechanisms that allow task difficulty, sensory input, and cognitive demands to be adjusted according to learner needs and experience levels. Such adaptability may be particularly valuable in rehabilitation and competency-based education contexts, where learners often exhibit diverse abilities and prior experiences.

Third, participants frequently described the interactive and experiential nature of AREduX as beneficial for understanding and applying concepts in realistic contexts. Participants indicated that actively engaging with simulation tasks supported understanding and retention more effectively than passive instructional approaches. These findings suggest that AREduX may be most effective when integrated into educational curricula as a supplementary tool for experiential learning, formative assessment, and reflective practice.

These design and educational recommendations are grounded in empirical findings [4-7] and demonstrate how AREduX's design principles can be leveraged to enhance engagement, learning outcomes, practical skill development, and transferability across rehabilitation and healthcare education domains.

Conclusion

Usability testing suggests that AREduX is a promising AR-based platform suggests that AREduX is a promising AR-based platform for healthcare education. AREduX provides healthcare professionals with immersive, real-time simulated experiences that may support experiential learning in a controlled and safe environment. Participants generally reported positive experiences with the simulation, and the results suggest that immersive AR experiences may have value for supporting the development of competencies relevant to healthcare practice, including empathy, communication, and clinical decision-making. These findings align with the broader simulation-based learning literature, which has demonstrated the potential of experiential learning approaches to support healthcare education and training.

A standout feature of AREduX is its ability to simulate selected sensory, cognitive, and functional challenges associated with dementia, offering healthcare professionals and caregivers the opportunity to directly experience the daily challenges faced by PLWD.

Participant feedback suggests that this immersive perspective-taking experience may contribute to greater understanding of patient challenges and support the development of empathy and communication skills.

The platform's modular design also suggests potential applications beyond dementia-focused training. Larger and more rigorous studies are required to evaluate the educational effectiveness of these applications and to determine whether the benefits observed in preliminary evaluations translate into measurable improvements in learning, clinical practice, or rehabilitation outcomes.

Future iterations of AREduX will refine its interface, expand task scenarios, and integrate more adaptive learning features, further solidifying AR as a transformative tool in healthcare innovation.
